# Metabolic Parameters and Emotionality Are Little Affected in G-Protein Coupled Receptor 12 (Gpr12) Mutant Mice

**DOI:** 10.1371/journal.pone.0042395

**Published:** 2012-08-07

**Authors:** Elisabeth Frank, Yizhen Wu, Naomi Piyaratna, William James Body, Peta Snikeris, Timothy South, Anna-Karin Gerdin, Mikael Bjursell, Mohammad Bohlooly-Y, Leonard Storlien, Xu-Feng Huang

**Affiliations:** 1 Schizophrenia Research Institute, Sydney, Australia; 2 School of Health Sciences, Illawarra Health and Medical Research Institute, University of Wollongong, Wollongong, Australia; 3 AstraZeneca R and D Mölndal, Mölndal, Sweden; 4 Boden Institute of Obesity, Nutrition, Exercise and Eating Disorders, University of Sydney, Sydney, Australia; Hosptial Infantil Universitario Niño Jesús, Ciberobn, Spain

## Abstract

**Background:**

G-protein coupled receptors (GPR) bear the potential to serve as yet unidentified drug targets for psychiatric and metabolic disorders. GPR12 is of major interest given its putative role in metabolic function and its unique brain distribution, which suggests a role in emotionality and affect. We tested Gpr12 deficient mice in a series of metabolic and behavioural tests and subjected them to a well-established high-fat diet feeding protocol.

**Methodology/Principal Findings:**

Comparing the mutant mice with wild type littermates, no significant differences were seen in body weight, fatness or weight gain induced by a high-fat diet. The Gpr12 mutant mice displayed a modest but significant lowering of energy expenditure and a trend to lower food intake on a chow diet, but no other metabolic parameters, including respiratory rate, were altered. No emotionality-related behaviours (assessed by light-dark box, tail suspension, and open field tests) were affected by the Gpr12 gene mutation.

**Conclusions/Significance:**

Studying metabolic and emotionality parameters in Gpr12 mutant mice did not reveal a major phenotypic impact of the gene mutation. Compared to previous results showing a metabolic phenotype in Gpr12 mice with a mixed 129 and C57Bl6 background, we suggest that a more pure C57Bl/6 background due to further backcrossing might have reduced the phenotypic penetrance.

## Introduction

Mood and metabolic disorders are often closely linked. A growing body of evidence shows that G-protein coupled receptors (GPR) play central roles in neuronal control of body functions, including psychiatric and metabolic disorders, and therefore present as targets for drug intervention for either [1]–[4]. GPRs comprise a large protein family of transmembrane receptors. Following extracellular stimulation, cell-surface GPRs activate intracellular signal transduction pathways that trigger important events related to cell differentiation, proliferation, development and survival [5]. Several GPRs have been de-orphanised in recent years, including the G protein-coupled Receptor 12 (GPR12).

The Gpr12 gene was initially cloned from rat pituitary [6] and later from human tissue [7]. It is expressed in several brain regions by both neurons and microglia [8], [9] as well as pituitary, ovary and testis tissue, but absent in other tissues [6]. Sphingosine-1-phosphate (S1p) [10], sphingosyl-phosphorylcholine (SPC) [11] and more recently Tyrosol [12] were reported as high affinity ligands for GPR12 amongst other GPRs. A phylogenetic tree places GPR12 in close proximity to cannabinoid receptors [13], [14]; of interest, because cannabinoid receptors are involved in regulation of both emotionality and metabolism [15], [16]. GPR12 was found to be highly abundant in the developing brain, particularly in postmitotic neurons during cerebral cortical development. Its occurrence shows a temporal pattern over development and studies suggested a role of GPR12 in neuronal differentiation, neuronal growth and the formation of synaptic contacts [9], [11]. It shows high similarity to GPR3 and GPR6, with overlapping ligand spectrum, but different ligand affinity in relation to differences in physiological function [11], [17], [18].

In adult mice, we and others found GPR12 in areas highly relevant to both emotionality and metabolism, including the cingulate cortex, hippocampus, habenular nucleus, nucleus accumbens, piriform cortex, septum and amygdala [1], [11]. In a previous study, we established a role of GPR12 in energy balance, as Gpr12 gene deficiency resulted in increased weight gain, decreased energy expenditure and dyslipidemia, with Gpr12 mutant mice showing symptoms of obesity [1].

In this study, we aimed to further examine the role of GPR12 in metabolic function, establish its influence on emotionality and relate our findings to plasma hormones which have been shown to have roles in both emotionality and metabolism [19]. Further, we explored a potential impact of various genetic backgrounds on the phenotype of a Gpr12 mutation, which was originally introduced in a mixed C57Bl/6 and 129/OlaHsd genetic background [1]. For this, we tested Gpr12 mutant mice that were further backcrossed into C57Bl/6 backgrounds from disparate animal providers compared to mice from our initial studies [1]. Animals were subject to a series of metabolic and behavioural tests as well as to a well established protocol of high-fat diet (HFD) feeding [20]. In line with previous results we found a modest effect of a Gpr12 deficiency on lowered energy expenditure. There was, however, no significant difference in body weight gain or composition, either under chow or HFD conditions, and no impact of a Gpr12 deficiency on the animals’ emotionality. Our data indicate a potential influence of the C57Bl/6 background with present results partially contrasting our previous findings in an earlier generation of Gpr12 deficient mice [1].

## Results

### Analysis of Gpr12 Mutant Mice Backcrossed for 6 Generations in C57Bl/6JOlaHsd – Swedish Studies

#### Metabolic parameters

**Figure 1:**
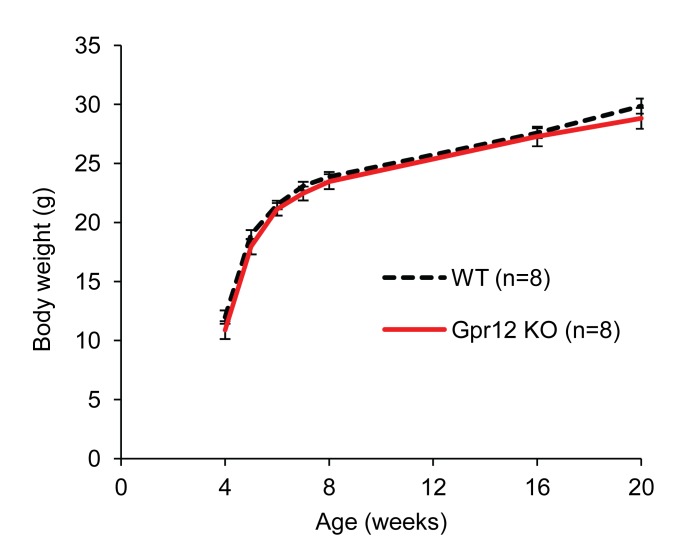
Body weight gain on lab chow. Body weight development in Gpr12 knockout (KO; n = 8) mice (red line) and wildtype (WT; n = 8) mice (dotted line) showed no difference at any measured time point.

Body weight at any measured time point (Figure 1) and body composition (Table 1) did not significantly differ between Gpr12 deficient (Gpr12 KO) and wildtype (WT) mice. In line with this, no significant differences were observed for absolute or relative body fat mass, body lean mass, bone mineral density or bone mineral content between Gpr12 KO and WT mice. Moreover, neither absolute nor relative (relative to body weight) energy intake was significantly different between the groups of mice (Table 1) when assessed by a fasting/refeeding study protocol.

**Table 1:** Body composition, food intake and body temperature of wild type (WT) and Gpr12 deficient (Gpr12 KO) mutant mice – Swedish study.

	WT	Gpr12 KO
**Body composition**		
Bone mineral density (mg/cm^2^)	43.61±0.37	43.19±0.44
Bone mineral content (g)	0.40±0.01	0.39±0.01
Rel. bone mineral content (mg/cm)	39.42±0.62	38.69±0.75
Body lean mass (g)	19.05±0.28	18.81±0.49
Rel. body lean mass (g/cm)	1.89±0.02	1.87±0.04
Body fat mass (g)	2.91±0.13	2.80±0.15
Rel. body fat mass (% of bw)	13.24±0.60	12.88±0.50
**Food intake**		
Absolute food intake (g/day)	7.58±0.36	7.94±0.21
Relative food intake (g/g bw)	0.33±0.02	0.34±0.01
Absolute energy intake (kcal/day)	23.3±1.11	24.5±0.65
Relative energy intake (kcal/g bw)	1.02±0.06	1.05±0.03
**Core rectal body temperature**		
Core temp. at room temp.	37.19±0.19	37.14±0.17
Core temp. at 5°C ambient temp.	37.51±0.13	37.28±0.15
n = 8 per group; bw Body weight		

**Figure 2:**
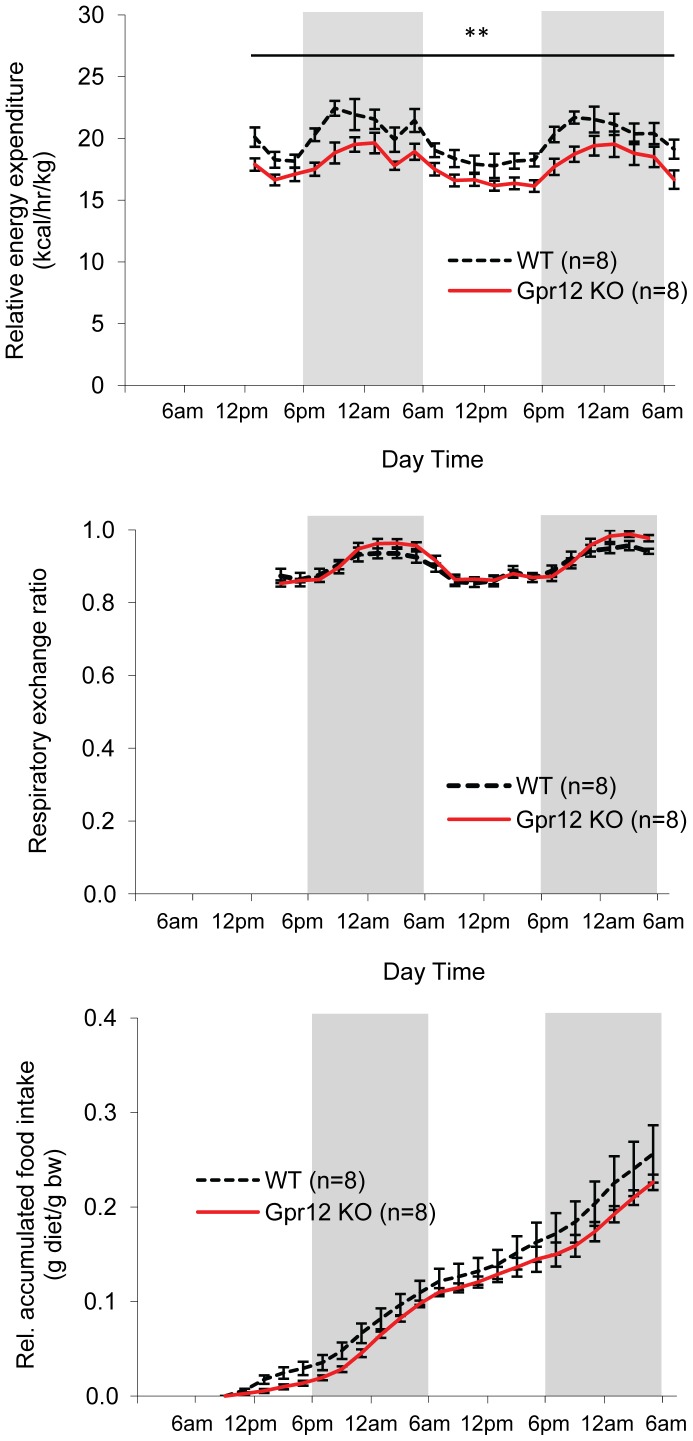
Energy expenditure. (A) Relative energy expenditure (EE), (B) respiratory exchange rate (RER) and (C) relative food intake (FI) of Gpr12 knockout (KO; n = 8) mice (red line) and wildtype (WT; n = 8) mice (dotted line). Whereas EE was significantly different between Gor12 KO and WT, RER and FI did not differ (2-way ANOVA with factors time and genotype; **p<0.01).

Gpr12 KO mice displayed, however, a significantly lower energy expenditure compared to WT mice (Figure 2A), but without a corresponding reduction of either core body temperature (Table 1) or locomotor activity (see behavioural tests below). Respiratory exchange ratio (Figure 2B) was not significantly different between Gpr12 KO and WT mice, indicating a similar energy source. Food intake showed a slight trend to be lower in Gpr12 KO mice when assessed simultaneously with energy expenditure (Figure 2C).

Core body temperature recorded at room temperature was not significantly different between Gpr12 KO and WT mice. Also when KO and WT mice were subjected to a 5°C ambient temperature for 45 minutes, no significant difference in core body temperature and therefore thermogenesis was observed (Table 1).

In the oral glucose tolerance test, no significant differences were observed in fasting blood glucose or blood glucose response to the orally administered glucose between Gpr12 KO and WT mice. Fasting insulin levels were, however, significantly higher (Table 2), whereas the insulin response to the glucose administration was not significantly different between Gpr12 KO and WT mice (data not shown).

**Table 2:** Blood chemistry and hepatic lipids of wild type (WT) and Gpr12 deficient (Gpr12 KO) mutant mice – Swedish study.

	WT	Gpr12 KO
**Levels after 5h fasting**		
Glucose (mM)	10.12±0.34	10.27±0.33
Plasma Insulin (ng/ml)	0.64±0.06	1.49±0.32*
n = 6 per group; *p<0.05		
**Plasma chemistry**		
Cholesterol (mM)	2.86±0.05	2.70±0.10
Triglyceride (mM)	0.90±0.05	1.03±0.11
NEFA (mM)	0.57±0.02	0.62±0.06
Leptin (ng/ml)	8.80±1.79	6.29±1.20
Adiponectin (nM)	165.4±13.5	152.8±17.2
**Hepatic lipid content**		
Hepatic lipid content (mg/g)	22.51±2.07	17.86±2.85
n = 8 per group; NEFA nonesterified fatty acids		

No significant differences were observed for blood plasma levels of triglycerides, total cholesterol, nonesterified fatty acids, leptin or adiponectin between Gpr12 KO and WT mice at 20 weeks of age. In liver biopsies hepatic triglyceride content was not significantly different between Gpr12 KO and WT mice (Table 2).

### Behavioural Tests

No significant differences between Gpr12 KO and WT mice were observed for any parameter in the open field locomotor activity analysis on either of the two test days (Table 3), including no significant differences in faecal pellet production during the experimental time (Table 3), indicating unaltered emotionality [21].

**Table 3:** Emotionality parameters of wild type (WT) and Gpr12 deficient (Gpr12 KO) mutant mice – Swedish study.

	WT	Gpr12 KO
**Behavioural parameters**		
- Open Field Test (day1)		
Ambulatory activity (beam breaks)	3648.6±126.6	4198.6±470.6
Faecal pellets (n)	6.0±0.9	5.9±0.6
- Open Field Test (day2)		
Ambulatory activity (beam breaks)	2805.4±212.6	2769.5±342.4
Faecal pellets (n)	5.1±0.8	6.8±1.0
n = 8 per group		

### Analysis of Gpr12 Mutant Mice re-derived in C57Bl/6ARC Mice – Australian Studies

#### Metabolic parameters

**Figure 3:**
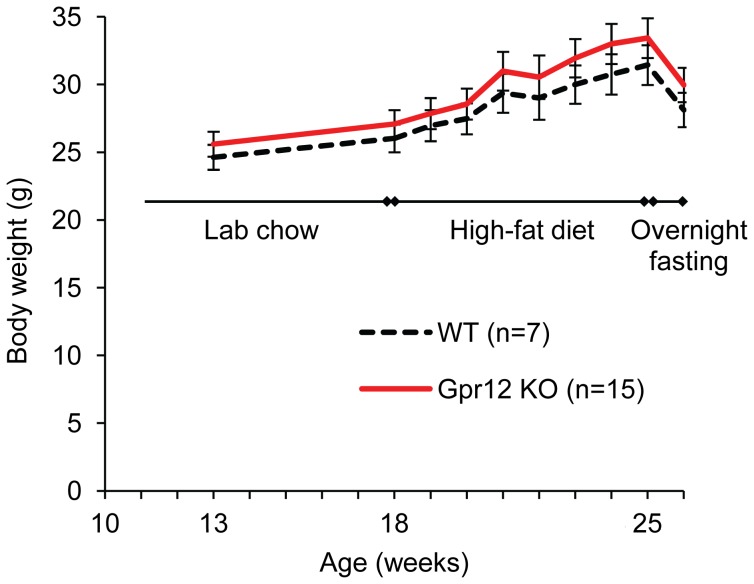
Body weight gain on high-fat diet. Body weight development in Gpr12 knockout (KO; n = 8) mice (red line) and wildtype (WT; n = 8) mice (dotted line) fed with lab chow, high-fat diet or after fasting showed no difference at any measured time point.

While fed with normal lab chow, no differences in body weight were found at 13 weeks or 18 weeks of age between Gpr12 KO and WT mice, despite a trend to higher weight in the Gpr12 KO group (Figure 3).

After onset of high-fat diet (HFD) feeding at 13 weeks of age, animals of all groups strongly increased their weight gain (p<0.01). There was, however, no significant interaction of weight increase over time and the respective genotypes, with also no difference in body weight at any time point between week 1 and week 7 of HFD feeding between Gpr12 KO and WT mice. When fasted overnight, all animals showed a significant weight loss (p<0.05), which was, however, not different between Gpr12 KO and WT mice.

Fasting levels of glucose, leptin and insulin did not differ between Gpr12 KO and WT mice (Table 4).

**Table 4:** Blood chemistry of wild type (WT) and Gpr12 deficient (Gpr12 KO) mutant mice – Australian study.

	WT	Gpr12 KO
**Levels after overnight fasting**		
Plasma Insulin (pg/ml)	42.4±21.3	38.5±11.2
Plasma Leptin (pg/ml)	270.7±69.8	318.6±116.5
Glucose (mM)	6.5±0.4	6.2±0.5
n = 7 for WT and n = 15 for Gpr12 KO		

### Behavioural Tests

For all behavioural parameters (Table 5), no significant differences between Gpr12 KO and WT animals were found, which included anxiety-related behaviours in the dark-light and open field test, depression-like parameters in the tail suspension test as well as locomotor activity in the dark-light box and open field test.

**Table 5:** Emotionality parameters of wild type (WT) and Gpr12 deficient (Gpr12 KO) mutant mice – Australian study.

Behavioural parameters		
- Dark-Light Box		
Time spent in light (s)	224.1±14.3	234.1±20.9
Distance moved (cm)	2773±153	2490±162
- Tail suspension Test		
Time immobile (s)	178.9±19.1	174.5±16.6
Latency immobility (s)	54.1±7.3	49.8±5.2
- Open Field Test		
Distance moved (cm)	10166±686	10809±414
Latency to inner zone (s)	34±12.6	41±7.2
Frequency inner zone (n)	63.2±6	54.7±6.9
n = 5 per group		

Heterozygous Gpr12 mice (n = 15) were subjects to the behavioural testing at the same time as Gpr12 KO and WT mice in order to determine if a reduction, rather than complete abrogation, of Gpr12 might have exposed a more subtle phenotypic effect. No differences between heterozygous Gpr12 mutant mice and either their Gpr12 KO or WT littermates were observed (Major test parameter results for heterozygous KO animals (see Table 5 for Gpr12 KO and WT results): Dark-Light Box, time spent in light (s): 206.2±11.7; Tail suspension test, time immobile (s): 186±5.1; Open Field test, distance moved (cm): 10689±274).

## Discussion

In this study, we examined the effects of a mutation in the G protein-coupled receptor 12 (Gpr12) gene on metabolic and emotionality parameters. With Gpr12 expression found in several brain areas highly relevant for emotionality [1], [9], we hypothesised that the Gpr12 gene might synergistically influence emotionality and metabolism. Studying Gpr12 deficient compared to wild type (WT) littermates from two different populations, we could, however, not detect any major differences in the studied metabolic parameters or emotionality-related behaviours.

In reporter cell lines, GPR12 was stated to constitutively activate both Gs and Gq pathways [22]. GPR12 stimulation can therefore positively regulate adenylyl cyclase to catalyse ATP to cAMP and further activate Protein kinase A. At the same time, it can activate phospholipase C to hydrolyse phosphatidylinositol bisphosphate to produce inositol trisphosphate (IP_3_) and diacylglycerol (DAG). Whereas IP_3_ will facilitate the release of calcium from intracellular stores, DAG recruits protein kinase C (PKC) and induces downstream phosphorylation [5]. Whereas both intracellular pathways have been shown to be critically involved in emotionality as well as in metabolic function, GPR12 has to date only been shown to significantly influence cAMP levels as required for meiotic arrest. We previously reported Gpr12 expression in areas highly relevant to both emotionality and metabolism, including the cingulated cortex, hippocampus, habenular nucleus, nucleus accumbens, piriform cortex, septum and amygdala [1], [11]. Whatever its function in these areas is, GPR12 does not appear to influence aspects of emotionality tested using the currently accepted techniques nor overall energy balance when on a more ‘pure’ C57/Bl6 background. Equally, our results are perhaps even more surprising given that GPR12 has been found to be highly abundant in the developing brain, particularly influencing neuronal differentiation [9], [11], based on which we expected a mutation in its gene to induce both metabolic and behavioural deficits.

### Metabolic Parameters

We have previously reported an early onset of increased weight gain, lower energy expenditure and increased body fat mass in Gpr12 mutant mice fed a normal chow diet and backcrossed for 4 generations towards C57Bl/6JOlaHsd [1]. Given the multiple examples of phenotype sensitivity of knockout mice from different sources [23], [24], we investigated potential influences of the Gpr12 mutation by varying genetic backgrounds with a new assessment set up in chow fed Gpr12 deficient mice backcrossed for 6 generations towards C57Bl/6JOlaHsd. In our previous study, we could show that a Gpr12 deficiency resulted in dyslipidemia and signs of obesity as a result of changed energy homeostasis. A significantly higher body weight was observed for Gpr12 deficient mice at both 5 and 11 weeks of age compared to WT mice, which was attributed to a decreased energy expenditure of the Gpr12 mutant mice [1]. In the present study when backcrossed only two to four more generations onto C57Bl/6 genetic background, we could not reproduce our previous findings of increased body weight gain or composition in Gpr12 deficient mutant when fed normal lab chow in either of the studied populations (Figures 1 and 3; Table 1). Backcrossing of our animals from the previously studied 4^th^ generation [1] to the 6^th^ generation in the presented Swedish studies increased the C57Bl6 background from 93.8% to 98.4%. Previous studies have shown that, likely due to epistatic interactions with the respective strain background, even small variation in purity can affect phenotypic penetrance [25]. We also tested heterozygous Gpr12 mutant mice (n = 12–15) to identify potential differences of reduction vs total ablation of GPR12 (data shown only for behavioural parameters). With no difference compared to either homozygous mutant or WT mice, this possibility was ruled out. The only difference, as observed previously [1] was modestly reduced energy expenditure in Gpr12 deficient mice (Figure 2A). The lack of effect on overall weight gain or body fat could not be explained by any overt differences in respiratory exchange rate (Figure 2B), body temperature at different ambient temperatures, or locomotor activity (Table 3). The trend towards slightly decreased food intake of Gpr12 KO mice (Figure 2C) as also observed previously [1] might account for why the reduced energy expenditure did not yield differences in weight gain. More subtle but relevant changes in these and other metabolic parameters cannot be ruled out.

To further investigate the potential impact of GPR12 on metabolism, we exposed Gpr12 KO and WT mice to a well established high-fat diet protocol (for composition see Table 6) [20], [26]. As with the outcomes on normal lab chow, we could not find any difference in body weight gain due to the genotype (Figure 3). We found, as previously reported, several diet resistant mice, which was, however, also independent of their genotype (data not shown). Body weight loss due to overnight fasting was also unaffected by the Gpr12 mutation. While fasting insulin levels were significantly higher in generation 6 C57Bl/6JOlaHsd backcrossed animals, they did not differ in re-derived C57Bl/6ARC mice, indicating possibly a subtle impact of the respective C57Bl/6 background. There were no other effects of genotype on other metabolic variables.

**Table 6:** High-fat diet composition – Australian study.

Ingredient	% Weight	% Energy/Kcal
**Total Carbohydrate**	**50**	**46**
Cornstarch	44	
Sucrose	6	
**Total Fat**	**19**	**38**
Lard	15	
Sunflower oil	4	
**Total Protein**	**18**	**16**
Gelatine	5	
Casein	13	
**Total others**	**13**	**0**
Fiber	5	
Minerals	7	
Vitamins	1	
Energy Density (ED) Kcal/g: 3.78		

### Emotionality-related Behaviour

In previous studies, GPR12 was demonstrated to be localised in areas highly involved in emotionality regulation and related behaviours, including the cingulate cortex, hippocampus and amygdala [1], [11]. The Gpr12 gene was found expressed in both neurons and microglia and involved in neurodevelopment [8], [9]. This indicated that deficits in GPR12 and its signalling, particularly during development as in mutant animals, could contribute to the development of psychopathologically relevant behaviours.

Genetic approaches in the form of targeted gene mutations have been used to study the pathophysiological impact of genetic loci on emotionality-related behavioural and neurochemical parameters to further understand the genetic basis of mood and metabolic disorders [27], [28]. Therefore, by conducting a series of behavioural tests specifically designed to examine anxiety-related and depression-like behaviour as well as locomotion, it was deemed to be possible to infer the role of a Gpr12 gene dysregulation in Gpr12 mutant mice.

When studying emotionality-related parameters in both the Swedish or Australian cohort of mice, we could not find any evidence for differences between Gpr12 mutant and WT mice. Considering that a reduction, rather than complete abrogation, of Gpr12 might reveal a phenotypic effect we also studied heterozygous Gpr12 mutant mice. In a preliminary study, heterozygous Gpr12 mutant mice exhibited trends towards increased depression-like behaviour in the tail suspension test (Piyaratna, unpublished). The tail suspension test has previously been shown to be relevant to uncover behavioural despair in genetically modified mice, with genetically engineered mice modelling psychopathologies exhibiting greater behavioural despair [27], [29]. However, despite testing a large number of heterozygous mutant mice (n = 15), no behavioural differences were seen in comparison to either Gpr12 KO or WT mice. Although the relatively small sample size in the Australian study is to be noted, its results are confirmed by the behavioural tests performed in the Swedish study as well as the results from heterozygous Gpr12 mutant mice,

With the important role of Gpr12 in early neurodevelopment and its distribution in both energy balance and emotionality relevant brain areas [1], [11], the totality of the present results is unexpected. Considering the high homology and overlapping ligand spectrum of GPR12 with other GPRs [11], [17], [18], compensatory responses, particularly of the highly homologous GPR3 and GPR6, remain as a potential explanation for the lack of effect of a Gpr12 mutation. Lack of expression data for these GPRs is a limitation of our studies.

Given our earlier more positive results [1] on 4 generation backcrossed mice, the current lack of differences may indicate a delicate interaction between the Gpr12 gene and other genes in the 129 compared to the C57Bl/6 genome. The importance of the genetic background into which a gene-targeted allele is introduced has previously been discussed to have the potential to influence the resulting phenotypes as well as their penetrance [24], [30]. Particularly for 129 and C57Bl/6 backgrounds, several studies have shown that the penetrance of phenotypic and hormonal characteristics of various gene deficiencies are dependant on the hosting mouse strains [24], [31]–[33]. Indeed, depending on the genetic background, the Gpr12 gene expression might be deviating from our previously reported pattern [1], and therefore potentially account for the relative penetrance of characteristics of a Gpr12 mutation in varying C57Bl/6 backgrounds.

Altogether, although we found a deficiency in energy expenditure in line with previous results, there was no effect of a Gpr12 mutation on any other metabolic and emotionality-related behavioural parameter studied. Considering its unique distribution in the brain and its involvement in neuronal differentiation [1], [9], [11], further studies accounting for the genetic backgrounds should address potential compensatory effects and the further potential of GPR12 as a drug target for psychopathology and metabolic disorders.

## Materials and Methods

Studies were carried out at AstraZeneca R&D Mölndal, Sweden, and the University of Wollongong, Australia, as indicated.

### Ethics Statement

All procedures involving experimental mice were performed in accordance with protocols approved by the local Animal Ethics Committee at the University of Gothenburg under the European Communities Council Directive and the Animal Ethics committee of the University of Wollongong under the Australian Code of Practice for the Care and Use of Animals for Scientific Purposes. All steps were taken to ameliorate suffering in all work involving our study animals, including regular health checks during housing and optimised, least impact experimental procedures as approved by the local ethics committees.

### Gpr12 Mutant Mice Backcrossed for 6 Generations in C57Bl/6JOlaHsd (Sweden)

Gpr12 mutant mice were obtained from Deltagen (San Carlos, USA) as described previously [1]. In Sweden, the mouse line was backcrossed for 6 generations on the C57bl/6JOlaHsd (Harlan, the Netherlands) inbred mouse strain before heterozygous intercross was performed to generate homozygous Gpr12 mutants (Gpr12 KO) and wild type litter mates (WT).

Male Gpr12 KO mice (n = 8) and WT littermates (n = 8) were subjected to a series of metabolic analyses and open field testing, as described below and previously [34], to understand how further backcrossing affects metabolic and behavioural phenotypes.

### Gpr12 Mutant Mice Re-derived in C57Bl/6ARC Mice (Australia)

Blastocytes of Gpr12 mutant mice backcrossed to C57Bl/6JOlaHsd for 8 generations were obtained and re-derived in C57Bl/6ARC (ARC, Australia) mice, and further backcrossed into C57Bl/6ARC for 4 generations at the animal facilities of the University of Wollongong, Australia, before creating homozygous Gpr12 mutants (Gpr12 KO), heterozygous Gpr12 mutants and wild type litter mates (WT) from a heterozygous intercross. Two separate sets of animals were used for the following experiments. Animals of the first set were fed a high-fat diet for 7 weeks commencing at the age of 18 weeks (Gpr12 KO n = 15, WT n = 7). The second set of animals was fed with lab chow ad libitum only and behavioural testing was performed as described below (Gpr12 KO n = 5, WT n = 5). Heterozygous Gpr12 animals (n = 12–15) have been included in the respective studies and tested at the same time, but with no difference to either homozygous or wildtype animals, data is only partially presented.

### Housing

At either facility, mice were housed under standard conditions (food and water ad libitum; 22±2°C; 12∶12 hour light-dark cycle). At the age of 4 weeks, mice were weaned and ear marked. Genotyping was performed using PCR on obtained ear tissue. For the Gpr12 gene, one primer was located upstream of the deleted region in the short arm (50-CT GTCTTTCCGTTGAAGAGGACAGG-30), a second primer located in exon 2 (50-TCACAGCAGATGAGGGTTCCTGAGC-30) and the third located in the targeting cassette (50-TTCAACAGACCTTGCATTCCTTTGG-30) [1].

### Metabolic Analyses (Sweden)

Body weight was recorded in chow (R3, Lactamin, Stockholm, Sweden) fed mice weekly from 4–8 weeks of age and then at 16 and 20 weeks of age. Body composition at 8 weeks of age was assessed by dual energy X-ray absorptiometry (DEXA) as previously described [34]. The mice were assessed at age 7 weeks in the comprehensive laboratory animal monitoring system (CLAMS, Columbus Instruments, Columbus, USA) over 48 consecutive hours at room temperature as described previously [34]. Food intake was assessed at 11 weeks of age over 48 hours by a fasting/re-feeding model, with 12 hour over-night fasting prior to experiment start as previously described [35]. Open field locomotor activity analysis was performed on two consecutive days at 8 weeks of age as described previously [34]. Core body temperature was recorded by a rectal probe, both at room temperature and also after exposure to a 5°C ambient temperature for 45 minutes. Fasting blood glucose, insulin and oral glucose tolerance was analysed in 5 hr diet deprived Gpr12 KO and WT mice at 12 weeks of age. Blood samples were collected from the tail vein before and 15, 30, 60, 90 and 120 minutes after oral glucose administration (2g/kg bw) to assess blood glucose (Accu-Chek, Roche Diagnostics, Manheim, Germany) and insulin (Crystal Chem, Downers Grove, USA).

Blood samples were taken at 20 weeks of age by cardiac puncture from isoflurane sedated non-fasted mice in EDTA coated tubes and plasma was separated by centrifugation and snap frozen in liquid nitrogen and subsequently stored in −80°C. Plasma levels of cholesterol, triglycerides, nonesterified fatty acids, leptin and adiponectin was assessed by commercial kit used previously by our group [36]. The liver was dissected, weight and hepatic triglyceride content determined as described previously [36].

### High-fat Diet (HFD) Feeding (Australia)

Body weight of all animals was measured at the age of 13 weeks and 18 weeks with animals being fed lab chow (Y.S. Feeds Pty Ltd., Young, Australia). Thereafter, animals were weighed weekly during HFD feeding as well as before and after fasting. Beginning with the age of 18 weeks, animals were fed with HFD (see Table 6 for diet composition) *ad libitum* for 7 weeks. After 7 weeks of HFD feeding, all animals were fasted overnight (no access to food; access to water *ad libitum*) and euthanised the next morning to measure fasting hormone plasma levels. Blood glucose was measured from a drop of tail blood using a Glucotrend Instrument (Roche, Germany) as reported previously [26]. Trunk blood was sampled in EDTA-coated tubes, supplemented with Aprotinin and centrifuged for 10 min at 4°C. Plasma was stored at −80°C until further analysis. Leptin and Insulin levels were measured using a 2-plex Luminex assay (Millipore, USA) on a LUMINEX 100 following the manufacturer’s instructions.

### Behavioural Tests (Sweden)

Assessment of open field locomotor activity was performed on two consecutive days to investigate exploratory behaviours in a novel environment as well as typical locomotor behaviours, and included recordings of horizontal activity, peripheral vs. central activity, rearing assessments and corner time, as previously described [34]. In short, the mice were placed in the centre of an open field (50×50 cm) at the start of the experiment and locomotor behaviours were assessed by recordings of beam breaks by infrared sensors over 60 min. After the experiment, the mice were removed to their home cages and the number of faecal boli produced over 60 min were noted.

### Behavioural Tests (Australia)

Behavioural testing commenced at the age of 8 weeks with all animals having only received lab chow ad libitum. All behavioural tests were carried out between 8am and 12pm on each testing day, with a two day test-interval. The recordings were analysed by an observer blind to the genotype using Ethovision (Noldus, Netherlands).

#### Dark-Light box (DaLi)

The DaLi box is an ethologically relevant approach-avoidance conflict test for anxiety-related behaviour, which is based on the natural conflict between the tendency of mice to explore a novel area and avoid brightly lit aversive areas [37], [38]. The test arena consisted of a dark black (15×25 cm, 5lux) and a bright white (25×25, 80lux) compartment, connected by a 5×5 cm gate. The apparatus was cleaned before each test session with water containing a detergent. The animal was placed into the dark compartment and could freely explore the arena for 10 min. Recorded behaviour was analysed using Ethovision. Time spent in the bright white area was used as an index for anxiety. The total locomotion in each compartment was assessed as locomotion parameter.

#### Tail suspension test (TST)

The TST belongs to the most widely used models for assessing depression-like behaviour in ethological, pharmacological and genetic mouse models [39], [40]. For the TST, animals were attached with scotch tape on the tail to the tail suspension device at a height of 30 cm, where they remained for 6 min as described previously [40]. The animal’s behaviour was videotaped and analysed by a trained observer blind to the genotype using Eventlog. The duration of and latency to immobility were taken as index of depression-like behaviour.

#### Open field test (OF)

Spontaneous and drug induced hyperlocomotion in the open field is used to study schizophrenia-related hyperactivity [41], [42]. Animals were exposed to a round open field arena (40 cm diameter, 20lx). The arena was cleaned with water containing a detergent before every animal. Animals were exposed for 30 min to the arena. Total distance travelled and time in the inner zone (16 cm diameter) were analysed using Ethovision.

### Statistical Analyses

Statistical analyses were performed using SPSS (version 17.0, SPSS Inc., Chicago, USA). The Kolmogorov-Smirnov test was used to analyse data for normal distribution. All data were found to be normally distributed. Repeated ANOVA was performed for repeated measures with the factors time and genotype. Independent groups were compared using a Student T-test. All data is expressed as means ± standard error of the mean (SEM), with significance accepted at p<0.05.
